# *N*-Monosubstituted Methoxy-oligo(ethylene glycol) Carbamate Ester Prodrugs of Resveratrol

**DOI:** 10.3390/molecules200916085

**Published:** 2015-09-03

**Authors:** Andrea Mattarei, Michele Azzolini, Mario Zoratti, Lucia Biasutto, Cristina Paradisi

**Affiliations:** 1 Department of Chemical Sciences, University of Padova, Via F. Marzolo, Padova 135131, Italy; E-Mail: cristina.paradisi@unipd.it; 2 Department of Biomedical Sciences, University of Padova, Viale G. Colombo, Padova 335121, Italy; E-Mails: michele.azzolini@gmail.com (M.A.); zoratti@bio.unipd.it (M.Z.); lucia.biasutto@cnr.it (L.B.); 3 CNR Neuroscience Institute, Viale G. Colombo, Padova 335121, Italy; 4 NÓOS srl, Via Campello sul Clitunno, Roma 3400181, Italy

**Keywords:** resveratrol, prodrugs, methoxy-oligo(ethylene glycol), poly(ethylene glycol), polyphenols, bioavailability, carbamate ester

## Abstract

Resveratrol is a natural polyphenol with many interesting biological activities. Its pharmacological exploitation *in vivo* is, however, hindered by its rapid elimination via phase II conjugative metabolism at the intestinal and, most importantly, hepatic levels. One approach to bypass this problem relies on prodrugs. We report here the synthesis, characterization, hydrolysis, and *in vivo* pharmacokinetic behavior of resveratrol prodrugs in which the OH groups are engaged in an *N*-monosubstituted carbamate ester linkage. As promoiety, methoxy-oligo(ethylene glycol) groups (m-OEG) (CH_3_–[OCH_2_CH_2_]_n_–) of defined chain length (n = 3, 4, 6) were used. These are expected to modulate the chemico-physical properties of the resulting derivatives, much like longer poly(ethylene glycol) (PEG) chains, while retaining a relatively low MW and, thus, a favorable drug loading capacity. Intragastric administration to rats resulted in the appearance in the bloodstream of the prodrug and of the products of its partial hydrolysis, confirming protection from first-pass metabolism during absorption.

## 1. Introduction

Evolution has endowed organisms with a powerful apparatus designed to detoxify “xenobiotic” substances which reach the cytoplasm [1,2,3]. These compounds tend to be hydrophobic/apolar to pass the cell membrane. In specific cells, such as enterocytes and hepatocytes, they are modified by the addition of polar groups (Phase I metabolism). One of the most common processes is the insertion of hydroxyl groups by P450 oxidases [4,5]. These hydroxyls are then targeted by conjugative enzymes of Phase II metabolism, in particular sulfotransferases [6,7] and uridine diphosphate-glucuronosyltransferases [8,9], to produce water-soluble, inactivated metabolites which can be re-exported via Organic Anion/Cation Transporters (OATPs) [10] and ATP-Binding Cassette (ABC)/Multi Drug Resistance (MDR) efflux transporters [11,12,13] and eliminated.

Plant polyphenols already possess, by definition, a number of hydroxyl groups. They are, thus, ready-made substrates for Phase II metabolism. Indeed, the modifications just mentioned have been long recognized and studied for representative members of this huge family of natural compounds, including resveratrol, which is of specific interest here. Rapid glucuronidation and sulfation in enterocytes, hepatocytes, and other cells (e.g., [14,15,16,17,18,19,20,21]) and re-export to the intestinal lumen by MDR proteins [15,22,23], or possibly OATPs [24], are thought to limit the potential impact of this celebrated “natural drug” *in vivo*. This is a pity, given the pleiotropic effects this dietary compound is reported to have in important pathophysiological respects (for recent reviews see, e.g., [25,26,27,28,29,30]). Possibly its most widely discussed activities are as an activator of the deacetylase SIRT1 and a repressor of inflammation, to which one might add its estrogen-like features (e.g., [31,32,33,34,35,36,37,38]). Pronounced metabolism may be an important reason why resveratrol’s effects are often unclear in human clinical studies [39,40,41,42,43,44]—a weakness shared with other polyphenols. A tool to increase resveratrol’s bioavailability and body levels may help dispel (or support) doubts, and, more importantly, allow the development of a full pharmacology of this compound.

One of the strategies used to prevent or delay drug metabolism and enhance bioavailability and effectiveness is based on prodrugs: the sites undergoing phase II conjugation, in our case the phenolic hydroxyls, are temporarily protected (–OH → –O–X–R) by removable groups during absorption, first pass through the liver and distribution, with final regeneration of the active principle following the removal of protective groups by chemical and/or enzyme-catalyzed hydrolysis.

Three features of the protecting group are most relevant: (1) the reactivity of the bond engaging the group to be protected must be suitable for the regeneration of the parent molecule in an appropriate time frame under the conditions prevailing *in vivo*; (2) the promoiety “capping” the reactive site ought to favor absorption and distribution to the desired body districts; and (3) the side-products resulting from its eventual dismantling ought to be innocuous or beneficial. 

This strategy is being actively applied to polyphenols (rev.s: [45,46]). A first family of resveratrol prodrugs was produced and tested [47], in which the phenolic OH groups are masked as *N,N*-disubstituted carbamate esters (–O–C(=O)–N(CH_3_)R), where R is a methoxy-poly(ethylene glycol)-350 (mPEG-350) or a butyl-glucosyl group. These derivatives are highly soluble in aqueous media but too stable to be used as prodrugs. *N*-monosubstituted carbamates are expected to undergo faster hydrolysis. However, the ideal promoiety remains to be chosen. Poly(ethylene glycol) chains are a strong candidate: their presence can increase aqueous solubility of hydrophobic drugs, prolong circulation time, slow down hydrolysis, and increase stability in the gastrointestinal tract [48,49,50,51,52,53]. This type of decoration is particularly valuable for protein/polypeptide drugs and drug-carrying liposomes (rev.s, e.g., [54,55,56,57,58,59]), which can be protected from phagocytosis and are less at risk of provoking an immune response. In general, polymer chains with MW in the range of a few to many kDa are employed for these modifications, as they perform better vis-à-vis hydration and immunogenicity.

Their size and size dispersion, however, complicate their chemistry in particular for purification and analytical aspects. Furthermore in the case of small molecules such as resveratrol the incorporation of such large structures in the prodrug means that the “drug loading capacity” of the construct, *i.e.* the amount of active principle associated with a given weight of precursor, is low. Siddalingappa *et al.* have recently reported micelle-forming constructs in which the resveratrol kernel was linked to 2- to 20-KDa PEG and PEG-PLA block copolymer chains via ether or carboxyester functionalities (succinyl linker) [60]. Micelle formation was concluded to enhance stability vis-à-vis ester hydrolysis. Constructs with MW up to 6.6 KDa were used injected intravenously into rats to study their pharmacokinetic behavior. We have explored the use of short-chain, defined-MW (monodispersed) methoxy-oligo(ethylene glycol) (m-OEG) as a convenient alternative to higher-MW PEGs [61]. These oligomeric moieties turned out to be useful in modulating physicochemical and absorption properties of derivatives, maintaining many properties of longer PEG chains with a more favorable drug loading capacity, and to be fully compatible with absorption from the gastrointestinal tract when linked to the stilbenoid kernel via acetal or ketal bond systems.

**Scheme 1:**
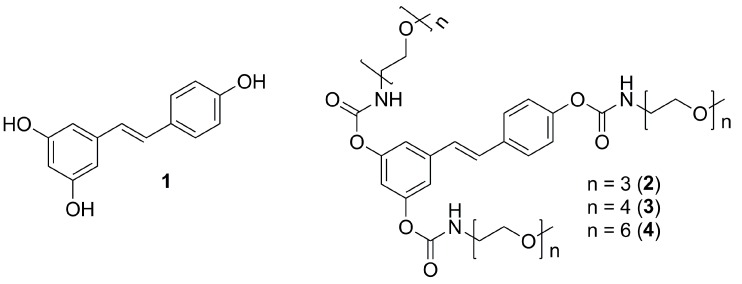
Resveratrol (**1**) and methoxy-oligo(ethylene glycol)-carbamate substituted prodrugs (**2**–**4**).

Herein we develop this approach reporting the synthesis and characterization of a small library of resveratrol prodrugs consisting of methoxy-oligo(ethylene glycol) amines linked to the resveratrol phenolic functions via the *N*-monosubstituted carbamate linkage (Scheme 1). Hydrolysis assays and pharmacokinetic studies in rats were then performed in order to evaluate stability, *in vivo*, absorption and metabolism of these new prodrugs.

## 2. Results and Discussion

### 2.1. Synthesis

**Scheme 2:**
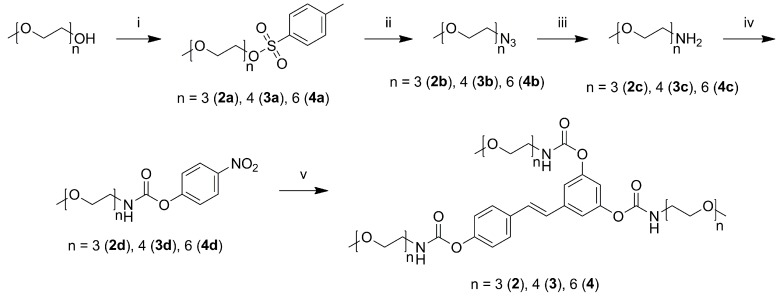
Synthesis of resveratrol *N*-monosubstituted carbamate ester prodrugs. Reagents and conditions: (i) TsCl, DMAP, Pyridine, DCM, 0 °C to r.t., 6 h; (ii) NaN_3_, H_2_O/Acetone 3:1, 75 °C, overnight_;_ (iii) PPh_3_, THF, r.t., 5 h, then H_2_O, 80 °C, overnight; (iv) bis-(4-nitrophenyl) carbonate, DMAP, ACN, 50 °C, 3 h; and (v) resveratrol, ACN, DMAP, 50 °C, 24 h.

Synthesis of *N*-monosubstituted carbamate esters is usually carried out in two steps: reaction of the desired primary amine with phosgene or its equivalent to give a reactive isocyanate derivative, followed by its coupling with the phenolic function [49]. These procedures tested on resveratrol led to low yields for the trisubstituted derivatives, probably due to the high reactivity of the isocyanate group promoting side reactions of polymerization entraining the stilbene double bond function. In this study we prepared *N*-monosubstituted resveratrol carbamate esters through conversion of the corresponding methoxy-oligo(ethylene glycol) amines (**2c**–**4c**) to the activated 4-nitrophenyl carbamates (Scheme 2). Isolation of the activated 4-nitrophenyl carbamate esters (**2d**–**4d**) followed by transesterification with resveratrol (**1**) provided the desired trisubstituted *N*-(methoxy-oligo(ethylene glycol)) carbamate prodrugs of resveratrol (**2**–**4**) in good yields (75%–88%) under mild conditions along with traces of di- and monosubstituted resveratrol conjugates, which were separated from the desired product by flash chromatography. Methoxy-oligo(ethylene glycol) amines (**2c**–**4c**) (key intermediates for the synthesis of **2**–**4**) were synthesized under mild conditions and with excellent yields (96%–98%) by Staudinger reduction of the corresponding methoxy-oligo(ethylene glycol) azides (**2b**–**4b**), in turn obtained by substitution of the tosylated methoxy-oligo(ethylene glycol) (**2a**–**4a**) with sodium azide (Scheme 2).

### 2.2. Hydrolysis Studies

The hydrolytic reactivity of the synthesized new resveratrol derivatives was tested in solutions mimicking gastric and intestinal pH and in blood. All turned out to be stable at pH values close to that of the human stomach (no reaction over 24 h at 37 °C in 0.1 N HCl), but underwent hydrolysis at near-neutral pH (at pH 6.8, representing intestinal pH) and in blood, at rates that may be suitable for use *in vivo*.

**Scheme 3:**
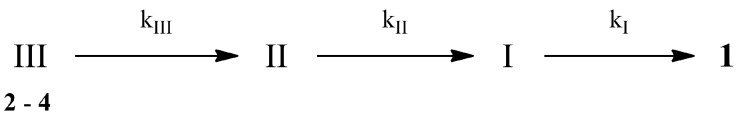
Kinetic scheme for stepwise hydrolysis of triprotected (**III**) derivatives **2**–**4** to resveratrol (**1**). **II** and **I** represent, each, the two possible isomeric derivatives resulting from hydrolysis of one (**II**) and two (**I**) carbamoyl linkages, respectively.

Kinetic analysis of the data was performed by assuming that hydrolysis to resveratrol occurred via consecutive losses of the three protecting groups in pseudo-first order processes and by considering each pair of isomeric intermediates (potentially) resulting from the first and second hydrolysis steps as a single species, *i.e.* the two monosubstituted and the two disubstituted intermediates were handled as species **I** and **II**, respectively (Scheme 3).

**Figure 1:**
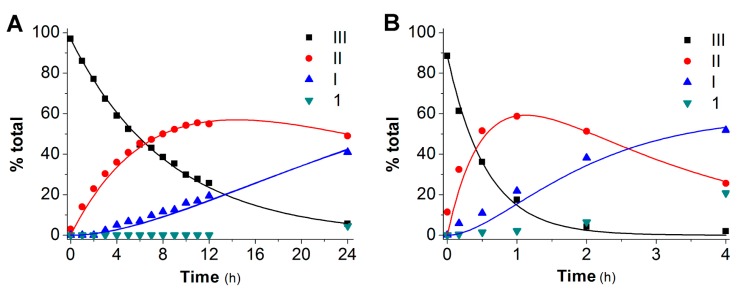
Hydrolysis of **3** in PBS 0.1 M, at pH 6.8, 37 °C (**A**) and in whole rat blood (**B**). Data are expressed as % of the initially-loaded compound. The fit is for pseudo-first order kinetics as outlined in Scheme 3 and accompanying text.

Figure 1 shows, as an example, the time course of the four species (**III**, **II**, **I,** and **1**) involved in the reaction of derivative **3** in PBS 0.1 M (panel **A**) and in whole rat blood (panel **B**). As hydrolysis proceeds resveratrol will eventually form with the kinetics dictated by the constants, up to 100% conversion. The fit of the experimental data was obtained using a set of equations analogous to those utilized by Kozerski *et al.* [62] and in our previous work [61].

**Figure 2:**
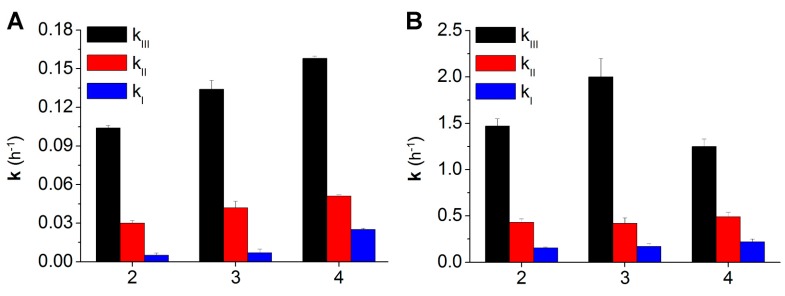
Hydrolysis rate constants (37 °C) for compounds **2**–**4** in PBS 0.1 M, at pH 6.8 (**A**) and in whole rat blood (**B**).

The full set of kinetic constants resulting from fits of this type for all the derivatives is presented in Table 1 and in Figure 2.

**Table 1:** Kinetic data obtained for hydrolysis of resveratrol derivatives **2**–**4** in aqueous PBS 0.1 M, at pH 6.8 and in rat blood at 37 °C.

Derivative	PBS 0.1 M, at pH 6.8, 37 °C				Blood			
	t_1/2_ (h)	k_III_ (h^−1^)	k_II_ (h^−1^)	k_I_ (h^−1^)	t_1/2_ (h)	k_III_ (h^−1^)	k_II_ (h^−1^)	k_I_ (h^−1^)
**2**	6.0	0.104 ± 0.002	0.030 ± 0.002	0.005 ± 0.002	0.5	1.47 ± 0.08	0.43 ± 0.04	0.155 ± 0.009
**3**	3.5	0.134 ± 0.007	0.042 ± 0.005	0.007 ± 0.003	0.3	2.0 ± 0.2	0.42 ± 0.06	0.17 ± 0.03
**4**	4.5	0.158 ± 0.002	0.051 ± 0.001	0.025 ± 0.001	0.5	1.25 ± 0.08	0.49 ± 0.05	0.22 ± 0.03

**Scheme 4:**
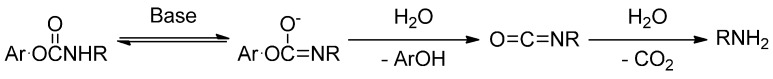
Mechanism of base-induced hydrolysis of carbamates **2**–**4**.

Hydrolysis of *N*-monosubstituted carbamate esters has been studied and reported in literature [63,64]. The stability of the *N*-monosubstituted carbamate group in acidic solution and its reactivity at higher pH values, are coherent with a mechanism which envisions the deprotonation of the amidic nitrogen ArO-C(O)-N*H*R′ followed by aryloxide elimination, thus regenerating the original phenolic functionality, ArOH. The isocyanate intermediate adds water and decomposes releasing the amine and carbon dioxide [63,64] (Scheme 4). As reported by L.W Dittert and T. Higuchi [64] the isocyanate intermediate formed during the hydrolysis of *N*-monosubstituted carbamate esters involves a lower energy of activation compared to the tetrahedral intermediate which is the only possible pathway for the OH^−^ catalyzed hydrolysis of *N,N*-disubstituted carbamate esters. This results in higher hydrolysis rates of *N-*monosubstituted carbamate esters under physiological pH conditions making this linkage more suitable for use as prodrug than the *N,N*-disubstituted carbamate ester bond.

The sensitivity of the carbamoyl group to pH ought to be taken into account when planning for an eventual oral administration of compounds of this type, since the pH of saliva in humans can normally vary from slightly acidic to slightly basic (pH 7.4) and hydrolytic enzymes are present. The residence time in the mouth would, in any case, be expected to be relatively short. The t_1/2_ of the compounds in blood (which has a slightly basic pH and hydrolytic enzymes) is in the order of 20–30 min. (Table 1).

The 4-h period over which the hydrolysis was monitored in whole blood was set by the stability of blood itself under the experimental conditions. It was in any case sufficient to compute the rate constants according to the model adopted. As hydrolysis proceeds resveratrol must eventually form with the kinetics dictated by the constants up to 100% conversion.

The data of Table 1 and Figure 2 clearly show that hydrolysis rates are much larger in blood, suggesting the involvement of enzyme-catalyzed hydrolysis. 

### 2.3. Pharmacokinetic Studies

All the *N*-monosubstituted methoxy-oligo(ethylene glycol) carbamate ester prodrugs were tested for their *in vivo* absorption and metabolism. Pharmacokinetic studies were performed with rats, and each compound was administered as a single intragastric bolus, in an equimolar dose/kg body weight (88 µmol/kg). Blood samples were taken at different time points, treated as described in the Materials and Methods section and analyzed. 

**Figure 3:**
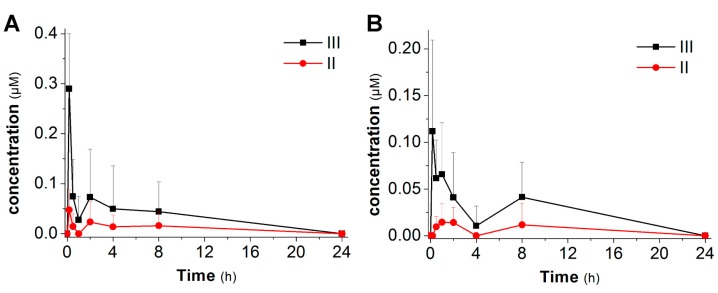
Blood pharmacokinetic profiles after intragastric administration of compounds **3** (**A**) and **4** (**B**) to rats. Data represent average values + standard deviation (an error bar of the same size in the negative direction is also implied but not shown for graphic clarity). N (number of PK experiments performed for each compound) = 3 in all cases.

Administration of compound **2** did not result in the appearance of detectable amounts of resveratrol, derivatives or any metabolites in blood samples, while measurable levels of stilbene derivatives (all compounds containing the stilbenic structure detected in blood, which can only originate from the administered prodrugs) were found in blood samples in the case of compounds **3** and **4**. The pharmacokinetic behavior of the prodrugs-absorption is bound to depend on the promoiety attached to the core compound. The fully protected and partially de-protected species for compounds **3** and **4** were consistently found in the samples taken, and the time course of their concentration is shown in Figure 3.

Both **3** and **4** were rapidly absorbed, peaking 10 min after their administration. In agreement with the results previously obtained with acetal prodrugs [61], the derivative with four units of ethylene glycol (**3**) was better absorbed than its counterpart with six units (0.3 µM *vs.* 0.1 µM). In both cases the di-substituted hydrolysis products were also present.

The results of PK studies confirm the observations reported in [61]: variations in the length of the methoxy-oligo(ethylene glycol) sequence can have an impact on the absorption of the prodrug. Thus, compounds **3** and **4**, which contain four and six units of ethylene glycol per phenolic oxygen, respectively, worked better than **2**, which contains three. The extent of absorption may be expected to be related to the affinity of the prodrugs for membranes if the step determining the rate of absorption is diffusion into the latter, *i.e.*, if the constructs are rather more hydrophilic than lipophilic. While we have not measured the distribution of the present compounds between water and an oily phase and their solubility, it is reasonable to presume that they may follow the same pattern as the corresponding acetal-based compounds of ref. [61].

## 3. Experimental Section 

### 3.1. Materials and Instrumentation

Resveratrol was purchased from Waseta Int. Trading Co. (Shangai, China). Other starting materials and reagents were purchased from Sigma-Aldrich (Milan, Italy), Fluka (Milan, Italy), Merck-Novabiochem (Milan, Italy), Riedel de Haen (Milan, Italy), J.T. Baker (Milan, Italy), Cambridge Isotope Laboratories Inc. (Rome, Italy), Acros Organics (Milan, Italy), Carlo Erba (Milan, Italy), and Prolabo (Milan, Italy) and were used as received. TLCs were run on silica gel supported on plastic (Macherey-Nagel Polygram^®^SIL G/UV_254_, silica thickness 0.2 mm, Duren, Germany) and visualized by UV detection. Flash chromatography was performed on silica gel (Macherey-Nagel 60, 230–400 mesh granulometry (0.063–0.040 mm)) under air pressure. The solvents were analytical or synthetic grade and were used without further purification. ^1^H-NMR spectra were recorded with a Bruker AC250F spectrometer operating at 250 MHz and a Bruker AVII500 spectrometer (Milan, Italy) operating at 500 MHz. Chemical shifts (δ) are given in ppm relative to the signal of the solvent. HPLC-UV analyses were performed with an Agilent 1290 Infinity LC System (Agilent Technologies, Milan, Italy), equipped with binary pump and a diode array detector (190–500 nm). HPLC/ESI-MS analyses and mass spectra were performed with a 1100 Series Agilent Technologies (Milan, Italy) system, equipped with binary pump (G1312A) and MSD SL Trap mass spectrometer (G2445D SL) with ESI source. ESI-MS positive spectra of reaction intermediates and final purified products were obtained from solutions in acetonitrile, eluting with a water:acetonitrile = 1:1 mixture containing 0.1% formic acid. High-resolution mass measurements were obtained using a Mariner ESI-TOF spectrometer (PerSeptive Biosystems, Framingham, MA, USA). HPLC/ESI-MS analysis was used to confirm the purity (>95%).

### 3.2. Hydrolysis Assays

#### 3.2.1. Hydrolysis under Physiological-Like Conditions

The chemical stability of all new compounds was tested in aqueous media imitating gastric (0.1 N HCl, NormaFix) and intestinal (0.1 M PBS buffer, at pH 6.8) pH conditions. A 5 µM solution of the compound was prepared from a 5 mM stock solution in DMSO, and incubated at 37 °C for 24 h; samples withdrawn at different times were analyzed by HPLC-UV. Hydrolysis products were identified by HPLC/ESI-MS analysis of selected samples. Non-linear curve fitting was performed using Origin 8.0 data analysis software, using the equations described in [61,62].

#### 3.2.2. Stability in Rodent Whole Blood

Rats were anesthetized and blood was withdrawn from the jugular vein, heparinised and transferred into tubes containing EDTA. Blood samples (1 mL) were spiked with 5 µM compound (dilution from a 5 mM stock solution in DMSO), and incubated at 37 °C for 4 h (the maximum period allowed by blood stability). Aliquots were taken after 10 min, 30 min, 1 h, 2 h and 4 h and treated as described below (blood sample treatment and analysis). Cleared blood samples were finally subjected to HPLC-UV analysis.

#### 3.2.3. Blood Sample Treatment and Analysis

Before starting the treatment, 4,4′-dihydroxybiphenyl was added as internal standard to a carefully-measured blood volume (25 µM final concentration). Blood was then stabilized with a freshly-prepared 10 mM solution of ascorbic acid (0.1 vol) and acidified with 0.6 M acetic acid (0.1 vol); after mixing, an excess of acetone (4 vol) was added, followed by sonication (2 min) and centrifugation (12,000× *g*, 7 min, 4 °C). The supernatant was finally collected and stored at −20 °C. Before analysis, acetone was allowed to evaporate at room temperature using a Univapo 150H (UniEquip, Martinsried, Germany) vacuum concentrator centrifuge, and up to 40 μL of CH_3_CN were added to precipitate residual proteins. After centrifugation (12,000× *g*, 5 min, 4 °C), cleared samples were directly subjected to HPLC-UV analysis. Metabolites and hydrolysis products were identified by comparison of chromatographic retention time with true samples.

The recovery yields of resveratrol and its metabolites have been reported previously [65,66]. For the new prodrugs the corresponding recoveries, expressed as ratio to the recovery of internal standard, were as follows: **2**: 0.914 ± 0.116 **3**: 0.903 ± 0.104; **4**: 0.967 ± 0.100. Recoveries of partially protected (disubstituted) derivatives were assumed to be the same as those of the corresponding fully substituted prodrug. Knowledge of these ratios allowed us to determine the unknown amount of analyte in a blood sample by measuring the internal standard recovered [54], and using the same calibration curve of resveratrol, since derivatives have the same absorption coefficient of resveratrol itself (y = 5.3085x, where y is the concentration of analyte and x is the integrated area of the HPLC-UV peak).

Since sample treatment includes an evaporation/concentration step, and there are no interfering peaks from the tissue matrix, LOD (limit of detection) and LOQ (limit of quantification) were determined relative to the analytical part of the method (HPLC-UV analysis) and were the same as resveratrol (*i.e.*, 0.04 µM and 0.12 µM, respectively; [65]).

### 3.3. Pharmacokinetic Studies

Derivatives **2**–**4** were administered to overnight-fasted male Wistar rats from the facility of the Department of Biomedical Sciences, University of Padova, as a single intragastric dose (88 µmol/kg, dissolved in 250 µL DMSO) delivered with a commercial stainless steel feeding tube (www.2biol.com) to have a precise starting time. Wistar rats are commonly used for such studies (e.g., [60]). Relatively young adults (3–5 months) were used in order to have a fully developed metabolism as well as a body weight (300–400 g) which would limit the amount of synthetic compound to be used. 

Blood samples were obtained by the tail bleeding technique: before drug administration, rats were anesthetized with isoflurane and the tip of the tail was cut off; blood samples (80–100 µL each) were then taken from the tail tip at different time points after drug administration. Blood was collected in heparinized tubes, kept in ice and treated as described above within 10 min. All experiments involving animals were performed with the permission and supervision of the University of Padova Ethical Committee for Experimentation on Animals (CEASA) and Central Veterinary Service, in compliance with Italian Law DL 116/92, embodying EU Directive 86/609. Significance in comparisons was assessed using the Wilcoxon Rank Test.

### 3.4. Synthesis

#### 3.4.1. General Procedure for the Preparation of Methoxy-oligo(ethylene glycol)-*p*-toluenesulfonates (**2a**–**4a**)

Pyridine (1.09 mL, 13.5 mmol, 2.0 eq.) and DMAP (1.65 g, 13.5 mmol, 2.0 eq.) were added to a solution of methoxy-oligo(ethylene glycol) (6.75 mmol, 1.0 eq.) in DCM (10 mL), and the mixture was stirred at 0 °C for 15 min. A solution of tosyl chloride (1.93 g, 10.1 mmol, 1.5 eq.) in DCM (10 mL) was then added dropwise and the reaction mixture was stirred at room temperature for 6 h. The resulting mixture was diluted in DCM (150 mL) and washed with 0.5 N HCl (100 mL). The aqueous layer was washed with DCM (5 × 75 mL) and all the organic fractions were collected, dried over MgSO_4_ and filtered. The solvent was evaporated under reduced pressure and the residue was purified by flash chromatography.

*2-(2-(2-Methoxyethoxy)ethoxy)ethyl 4-methylbenzenesulfonate* (**2a**). Purified by flash chromatography using DCM/EtOAc 8:2 as eluent. 95% yield as a colourless oil. ^1^H-NMR (250 MHz, CDCl_3_) δ (ppm): 2.43 (s, 3H, Ar–C*H_3_*), 3.35 (s, 3H, –O–C*H_3_*), 3.49–3.66 (m, 10H, 2 × –O–C*H_2_*–C*H_2_*–O– + –O–C*H_2_*–), 4.14 (t, 2H, Ts–C*H_2_*–, ^3^*J*_H–H_ = 5.75 Hz), 7.32 (d, 2H, Ar–*H*, ^3^*J*_H–H_ = 8.25 Hz), 7.77 (d, 2H, Ar–*H*, ^3^*J*_H–H_ = 8.25 Hz); ^13^C-NMR (62.9 MHz, CDCl_3_) δ (ppm): 144.7, 132.9, 129.7, 127.9, 71.8, 70.6, 70.5, 70.4, 69.2, 68.6, 58.9, 21.6; ESI-MS (ion trap): *m*/*z* 337 [M + H_2_O + H]^+^. HRMS (ESI+): *m/z* 319.1222 [M + H]^+^, calcd for C_14_H_23_O_6_S: 319.1215.

*2,5,8,11-Tetraoxatridecan-13-yl 4-methylbenzenesulfonate* (**3a**). Purified by flash chromatography using DCM/Acetone 8:2 as eluent. 94% yield as a colourless oil. ^1^H-NMR (250 MHz, CDCl_3_) δ (ppm): 2.41 (s, 3H, Ar–C*H_3_*), 3.33 (s, 3 H, –O–C*H_3_*), 3.48–3.67 (m, 14 H, 3 × –O–C*H_2_*–C*H_2_*–O– + –O–C*H_2_*–), 4.12 (t, 2H, Ts–C*H_2_*–, ^3^*J*_H–H_ = 4.90 Hz), 7.30 (d, 2H, Ar–*H*, ^3^*J*_H–H_ = 8.30 Hz), 7.76 (d, 2H, Ar–*H*, ^3^*J*_H–H_ = 8.00 Hz); ^13^C-NMR (62.9 MHz, CDCl_3_) δ (ppm): 144.7, 132.8, 129.7, 127.8, 71.8, 70.6, 70.4, 70.4, 70.4, 70.3, 69.1, 68.5, 58.9, 21.5; ESI-MS (ion trap): *m*/*z* 381 [M + H_2_O + H]^+^. HRMS (ESI+): *m/z* 363.1463 [M + H]^+^, calcd for C_16_H_27_O_7_S: 363.1477.

*2,5,8,11,14,17-Hexaoxanonadecan-19-yl 4-methylbenzenesulfonate* (**4a**). Purified by flash chromatography using DCM/Acetone 6.5:3.5 as eluent. 98% yield as a colourless oil. ^1^H-NMR (250 MHz, CDCl_3_) δ (ppm): 2.35 (s, 3H, Ar–C*H_3_*), 3.27 (s, 3H, –O–C*H_3_*), 3.42–3.60 (m, 22H, 2 × –O–C*H_2_*–C*H_2_*–O– + –O–C*H_2_*–), 4.05 (t, 2H, Ts–C*H_2_*–, ^3^*J*_H–H_ = 5.00 Hz), 7.25 (d, 2H, Ar–*H*, ^3^*J*_H–H_ = 7.93 Hz), 7.70 (d, 2H, Ar–*H*, ^3^*J*_H–H_ = 8.34 Hz); ^13^C-NMR (62.9 MHz, CDCl_3_) δ (ppm): 144.6, 132.7, 129.6, 127.7, 71.7, 70.5, 70.4, 70.4, 70.3, 70.3, 70.3, 69.1, 68.4, 58.8, 21.4; ESI-MS (ion trap): *m*/*z* 451 [M + H]^+^. HRMS (ESI+): *m/z* 451.2008 [M + H]^+^, calcd for C_20_H_35_O_9_S: 451.2002.

#### 3.4.2. General Procedure for the Preparation of Methoxy-oligo(ethylene glycol)-azides (**2b**–**4b**)

Sodium azide (10.72 g, 0.165 mol, 5.0 eq.) were added to a solution of methoxy-oligo(ethylene glycol)-*p*-toluenesulfonate (**2a**–**4a**) (33 mmol, 1.0 eq.) in a water/acetone solution (1:3, 65 mL), and the mixture was stirred at 75 °C overnight. The mixture was then diluted in DCM (250 mL) and washed with water (250 mL). The aqueous layer was washed with DCM (5 × 100 mL) and all the organic fractions were collected, dried over MgSO_4_ and filtered. The solvent was evaporated under reduced pressure and the residue was purified by flash chromatography.

*1-Azido-2-(2-(2-methoxyethoxy)ethoxy)ethane* (**2b**). Purified by flash chromatography using DCM/Acetone 9:1 as eluent. 99% yield as a colourless oil. ^1^H-NMR (250 MHz, CDCl_3_) δ (ppm): 3.32–3.36 (m, 5H, –O–C*H_3_* + –O–CH_2_–C*H_2_*–N_3_), 3.48–3.58 (m, 2H, –O–C*H_2_*–CH_2_–N_3_), 3.48–3.58 (m, 8H, 2 × –O–C*H_2_*–C*H_2_*–O–); ^13^C-NMR (62.9 MHz, CDCl_3_) δ (ppm): 71.7, 70.5, 70.5, 70.4, 69.9, 58.8, 50.5; ESI-MS (ion trap): *m*/*z* 190 [M + H]^+^. HRMS (ESI+): *m*/*z* 190.1207 [M + H]^+^, calcd for C_7_H_16_N_3_O_3_: 190.1192.

*13-Azido-2,5,8,11-tetraoxatridecane* (**3b**). Purified by flash chromatography using DCM/Acetone 85:15 as eluent. 97% yield as a colourless oil. ^1^H–NMR (250 MHz, CDCl_3_) δ (ppm): 3.32–3.36 (m, 5H, –O–C*H_3_* + –O–CH_2_–C*H_2_*–N_3_), 3.48–3.52 (m, 2H, –O–C*H_2_*–CH_2_–N_3_), 3.58–3.65 (m, 12H, 3 × –O–C*H_2_*–C*H_2_*–O–); ^13^C-NMR (62.9 MHz, CDCl_3_) δ (ppm): 71.8, 70.5, 70.5, 70.5, 70.4, 70.3, 69.9, 58.9, 50.5; ESI-MS (ion trap): *m*/*z* 234 [M + H]^+^. HRMS (ESI+): *m*/*z* 234.1460 [M + H]^+^, calcd for C_8_H_20_N_3_O_4_: 234.1454.

*19-Azido-2,5,8,11,14,17-hexaoxanonadecane* (**4b**). Purified by flash chromatography using DCM/Acetone 8:2 as eluent. 96% yield as a colourless oil. ^1^H-NMR (250 MHz, CDCl_3_) δ (ppm): 3.28–3.32 (m, 5H, –O–C*H_3_* + –O–CH_2_–C*H_2_*–N_3_), 3.43–3.48 (m, 2H, –O–C*H_2_*–CH_2_–N_3_), 3.53–3.61 (m, 20H, 5 × –O–C*H_2_*–C*H_2_*–O–); ^13^C-NMR (62.9 MHz, CDCl_3_) δ (ppm): 71.6, 70.4, 70.4, 70.3, 70.3, 70.3, 70.3, 70.3, 70.2, 69.7, 58.7, 50.4; ESI-MS (ion trap): *m*/*z* 322 [M + H]^+^. HRMS (ESI+): *m*/*z* 322.1989 [M + H]^+^, calcd for C_13_H_28_N_3_O_6_: 322.1978.

#### 3.4.3. General Procedure for the Preparation of Methoxy-oligo(ethylene glycol)-amines (**2c**–**4c**)

Triphenylphosphine (10.88 g, 41.5 mmol, 1.25 eq.) in anhydrous THF (25 mL) was added dropwise to a solution of methoxy-oligo(ethylene glycol)-azide (**2b**–**4b**) (33.0 mmol, 1.0 eq.) in anhydrous THF (25 mL), and the solution was stirred at RT for 5 h. Distilled water (20 mL) was then added and the mixture was heated under reflux (80 °C) and vigorously stirred overnight. The resulting mixture was evaporated under reduced pressure and the residue was purified by flash chromatography.

*2-(2-(2-Methoxyethoxy)ethoxy)ethanamine* (**2c**). Purified by flash chromatography using DCM/MeOH = 9:1 (+ 1% Et_3_N) as eluent. 97% yield as a pale yellow oil. ^1^H-NMR (250 MHz, CDCl_3_) δ (ppm): 1.57 (s, 2H, –CH_2_–N*H_2_*), 2.79 (t, 2H, ^3^*J*_H–H_ = 5.25 Hz, –CH_2_–C*H_2_*–NH_2_), 3.31 (s, 3H, –O–C*H_3_*), 3.41–3.51 (m, 4H, –O–CH_2_–C*H_2_*–O– + –O–C*H_2_*–CH_2_–NH_2_), 3.52–3.63 (m, 6H, –O–C*H_2_*–C*H_2_*–O– + –O–C*H_2_*–CH_2_–O–); ^13^C-NMR (62.9 MHz, CDCl_3_) δ (ppm): 73.2, 71.6, 70.3, 70.3, 70.0, 58.8, 41.5; ESI-MS (ion trap): *m*/*z* 164 [M + H]^+^. HRMS (ESI+): *m*/*z* 164.1275 [M + H]^+^, calcd for C_7_H_18_NO_3_: 164.1287.

*2,5,8,11-Tetraoxatridecan-13-amine* (**3c**). Purified by flash chromatography using DCM/MeOH = 9:1 (+ 1% Et_3_N) as eluent. 96% yield as a pale yellow oil. ^1^H-NMR (250 MHz, CDCl_3_) δ (ppm): 1.54 (s, 2H, –CH_2_–N*H_2_*), 2.71 (t, 2H, ^3^*J*_H–H_ = 5.25 Hz, –CH_2_–C*H_2_*–NH_2_), 3.22 (s, 3H, –O–C*H_3_*), 3.34–3.41 (m, 4H, –O–CH_2_–C*H_2_*–O– + –O–C*H_2_*–CH_2_–NH_2_), 3.45–3.51 (m, 10H, 2 × –O–C*H_2_*–C*H_2_*–O– + –O–C*H_2_*–CH_2_–O–); ^13^C-NMR (62.9 MHz, CDCl_3_) δ (ppm): 40.7, 39.3, 38.0, 37.9, 37.9, 37.9, 37.6, 26.4, 9.1; ESI-MS (ion trap): *m*/*z* 208 [M + H]^+^. HRMS (ESI+): *m*/*z* 208.1552 [M + H]^+^, calcd for C_9_H_22_NO_4_: 208.1549.

*2,5,8,11,14,17-Hexaoxanonadecan-19-amine* (**4c**). Purified by flash chromatography using DCM/MeOH = 9:1 (+ 1% Et_3_N) as eluent. 98% yield as a pale yellow oil. ^1^H-NMR (250 MHz, CDCl_3_) δ (ppm): 1.78 (s, 2H, –CH_2_–N*H_2_*), 2.76 (t, 2H, ^3^*J*_H–H_ = 5.25 Hz, –CH_2_–C*H_2_*–NH_2_), 3.26 (s, 3H, –O–C*H_3_*), 3.39–3.46 (m, 4H, –O–CH_2_–C*H_2_*–O– + –O–C*H_2_*–CH_2_–NH_2_), 3.51–3.56 (m, 18H, 4 × –O–C*H_2_*–C*H_2_*–O– + –O–C*H_2_*–CH_2_–O–); ^13^C-NMR (62.9 MHz, CDCl_3_) δ (ppm): 72.8, 71.6, 70.3, 70.3, 70.3, 70.2, 70.2, 70.2, 70.0, 58.7, 41.4; ESI-MS (ion trap): *m*/*z* 296 [M + H]^+^. HRMS (ESI+): *m*/*z* 296.2077 [M + H]^+^, calcd for C_13_H_30_NO_6_: 296.2073.

#### 3.4.4. General Procedure for the Preparation of Activated 4-Nitrophenyl Methoxy-oligo(ethylene glycol) Urethanes (**2d**–**4d**)

A solution of methoxy-oligo(ethylene glycol) amine (**2c**–**4c**) (8.2 mmol, 1.0 eq.) and DMAP (2.00 g, 16.4 mmol, 2.0 eq.) in acetonitrile (15 mL) was added dropwise to a solution of bis(4-nitrophenyl) carbonate (2.74 g, 9.0 mmol, 1.1 eq.) in acetonitrile (15 mL) and the resulting solution was stirred at 50 °C for 3 h. The reaction mixture was then diluted in DCM (150 mL) and washed with 0.5 N HCl (100 mL). The aqueous layer was washed with DCM (5 × 100 mL) and all the organic fractions were collected, dried over MgSO_4_ and filtered. The solvent was evaporated under reduced pressure and the residue was purified by flash chromatography.

*4-Nitrophenyl (2-(2-(2-methoxyethoxy)ethoxy)ethyl)carbamate* (**2c**). Purified by flash chromatography using DCM/Acetone = 9:1 as eluent. 87% yield as a pale yellow oil. ^1^H-NMR (250 MHz, CDCl_3_) δ (ppm): 3.31 (s, 3H, –O–C*H_3_*), 3.37–3.43 (m, 2H, –O–CH_2_–C*H_2_*–NH–), 3.49–3.63 (m, 10H, 2 × –O–C*H_2_*–C*H_2_*–O– + –O–C*H_2_*–CH_2_–NH–), 6.06 (t, 1H, –N*H*–, ^3^*J*_H–H_ = 5 Hz), 7.25 (d, 2H, Ar–*H*, ^3^*J*_H–H_ = 9.25 Hz), 8.15 (d, 2H, Ar–*H*, ^3^*J*_H–H_ = 9.25 Hz); ^13^C-NMR (62.9 MHz, CDCl_3_) δ (ppm): 155.9, 153.1, 144.3, 124.8, 121.8, 71.6, 70.2, 70.2, 69.9, 69.3, 58.7, 40.8; ESI-MS (ion trap): *m*/*z* 329 [M + H]^+^. HRMS (ESI+): *m/z* 329.1357 [M + H]^+^, calcd for C_14_H_21_N_2_O_7_: 329.1349.

*4-Nitrophenyl 2,5,8,11-tetraoxatridecan-13-ylcarbamate* (**3c**). Purified by flash chromatography using DCM/Acetone = 85:15 as eluent. 85% yield as a pale yellow oil. ^1^H-NMR (250 MHz, CDCl_3_) δ (ppm): 3.17 (s, 3H, –O–C*H_3_*), 3.24–3.30 (m, 2H, –O–CH_2_–C*H_2_*–NH–), 3.35–3.50 (m, 14H, 3 × –O–C*H_2_*–C*H_2_*–O– + –O–C*H_2_*–CH_2_–NH–), 6.21 (t, 1H, –N*H*–, ^3^*J*_H–H_ = 5 Hz), 7.15 (d, 2H, Ar–*H*, ^3^*J*_H–H_ = 9.25 Hz), 8.03 (d, 2H, Ar–*H*, ^3^*J*_H–H_ = 9.25 Hz); ^13^C-NMR (62.9 MHz, CDCl_3_) δ (ppm): ^13^C-NMR (63 MHz, CDCl_3_) δ (ppm) 156.1, 153.2, 144.3, 124.9, 121.9, 71.7, 70.4, 70.3, 70.2, 70.1, 69.4, 58.7, 41.0; ESI-MS (ion trap): *m*/*z* 373 [M + H]^+^. HRMS (ESI+): *m/z* 373.1619 [M + H]^+^, calcd for C_16_H_25_N_2_O_8_: 373.1611.

*4-Nitrophenyl 2,5,8,11,14,17-hexaoxanonadecan-19-ylcarbamate* (**4c**). Purified by flash chromatography using DCM/Acetone gradient from 8:2 to 6:4 as eluent. 84% yield as a pale yellow oil. ^1^H-NMR (250 MHz, CDCl_3_) δ (ppm): 3.30 (s, 3H, –O–C*H_3_*), 3.37–3.50 (m, 4H, –O–C*H_2_*–C*H_2_*–NH–), 3.55–3.61 (m, 20H, 5 × –O–C*H_2_*–C*H_2_*–O–), 6.04 (t, 1H, –N*H*–, ^3^*J*_H–H_ = 5 Hz), 7.26 (d, 2H, Ar–*H*, ^3^*J*_H–H_ = 9.25 Hz), 8.17 (d, 2H, Ar–*H* , ^3^*J*_H–H_ = 9.25 Hz); ^13^C-NMR (62.9 MHz, CDCl_3_) δ (ppm): 125.8, 124.9, 121.8, 115.5, 71.6, 70.3, 70.3, 70.3, 70.2, 70.0, 69.3, 58.8, 40.9; ESI-MS (ion trap): *m*/*z* 461 [M + H]^+^. HRMS (ESI+): *m/z* 461.2137 [M + H]^+^, calcd for C_13_H_30_NO_6_: 461.2135.

#### 3.4.5. General Procedure for the Preparation of 3,4′,5-*N*-Monosubstituted-methoxy-oligo(ethylene glycol) Resveratrol Carbamate Esters (**2**–**4**)

A solution of resveratrol (0.24 g, 1.1 mmol, 1.0 eq.) and DMAP (0.52 g, 4.2 mmol, 4.0 eq.) in ACN (15 mL) was added to a solution of the activated 4-nitrophenyl methoxy-oligo(ethylene glycol) urethane (**2c**–**4c**) (4.8 mmol, 4.5 eq) in ACN (5 mL) and the resulting mixture was allowed to react under vigorous stirring at 50 °C for 24 h. The reaction mixture was diluted with DCM (150 mL) and washed with 0.5 N HCl (100 mL). The aqueous layer was washed with DCM (5 × 75 mL) and all the organic fractions were collected, dried over MgSO_4_ and filtered. The solvent was evaporated under reduced pressure and the residue was purified by flash chromatography.

*(E)-5-(4-(2-(2-(2-Methoxyethoxy)ethoxy)ethyl)carbamate)-1,3-phenylene bis((2-(2-(2-methoxyethoxy) ethoxy)ethyl)carbamate)* (**2**). Purified by flash chromatography using DCM/Acetone = 6.5:3.5 as eluent. 75% yield as a pale yellow oil. ^1^H-NMR (250 MHz, CDCl_3_) δ (ppm): 3.08 (m, 9 H, 3 × –O–C*H_3_*), 3.39 (s, 9H, 3 × –O–C*H_3_*) 3.42–3.69 (m, 36H, 6 × –O–C*H_2_*–C*H_2_*–O– + 3 × –O–C*H_2_*–C*H_2_*–NH–), 5.80 (m, 3H, 3 × –N*H*–), 6.86 (t, 1H, ^4^*J*_H–H_ = 2.0 Hz, H–4), 6.90–7.12 (m, 6H, H–2, H–3′, H–5′, H–6, H–7, H–8), 7.45 (d, 2H, ^3^*J*_H–H_ = 8.75 Hz, H–2′, H–6′); ^13^C–NMR (62.9 MHz, CDCl_3_) δ (ppm): 154.6, 154.3, 151.6, 150.7, 139.1, 133.9, 129.2, 127.4, 127.1, 121.8, 116.3, 114.3, 71.8, 70.5, 70.2, 70.2, 69.8, 59.0, 40.9; ESI-MS (ion trap): m/z 818 [M + Na]^+^. HRMS (ESI+): *m/z* 796.3873 [M + H]^+^, calcd for C_38_H_58_N_3_O_15_: 796.3868.

*(E)-5-(4-(5,8,11-Tetraoxatridecan-13-ylcarbamate)styryl)-1,3-phenylene bis(2,5,8,11-tetraoxatridecan -13-ylcarbamate)* (**3**). Purified by flash chromatography using DCM/Acetone = 5:5 as eluent. 88% yield as a colourless oil. ^1^H-NMR (250 MHz, CDCl_3_) δ (ppm): 3.35 (s, 9H, 3 × –O–C*H_3_*), 3.41–3.56 (m, 12H, 3 × –O–C*H_2_*–C*H_2_*–NH–), 3.60–3.68 (m, 36H, 9 × –O–C*H_2_*–C*H_2_*–O–), 5.81–5.88 (m, 3H, 3 × –N*H*–), 6.80–7.14 (m, 7H, H–2, H–4, H–3′, H–5′, H–6, H–7, H–8), 7.44 (d, 2H, ^3^*J*_H–H_ = 8.75 Hz, H–2′, H–6′); 13C–NMR (62.9 MHz, CDCl_3_) δ (ppm): 154.5, 154.2, 151.5, 150.6, 138.9, 133.7, 129.1, 127.3, 126.9, 121.7, 116.2, 114.2, 71.7, 70.4, 70.3, 70.3, 70.1, 70.0, 69.6, 58.8, 40.8; ESI-MS (ion trap): *m*/*z* 928 [M + H]^+^. HRMS (ESI+): *m/z* 928.4669 [M + H]^+^, calcd for C_44_H_70_N_3_O_18_: 928.4654.

*(E)-5-(4-(2,5,8,11,14,17-Hexaoxanonadecan-19-ylcarbamate)styryl)-1,3-phenylene bis(2,5,8,11,14,17-hexaoxanonadecan-19-ylcarbamate)* (**4**). Purified by flash chromatography using DCM/Acetone from 4:6 to 2:8 as eluent. 76% yield as a colourless oil. ^1^H-NMR (250 MHz, CDCl_3_) δ (ppm): 3.33 (s, 9H, 3 × –O–C*H_3_*), 3.38–3.51 (m, 12H, 3 × –O–C*H_2_*–C*H_2_*–NH–), 3.57–3.68 (m, 60H, 15 × –O–C*H_2_*–C*H_2_*–O–), 5.80–5.86 (m, 3H, 3 × –N*H*–), 6.80–7.12 (m, 7H, H–2, H–4, H–3′, H–5′, H–6, H–7, H–8), 7.41 (d, 2H, ^3^*J*_H–H_ = 8.75 Hz, H–2′, H–6′); ^13^C-NMR (62.9 MHz, CDCl_3_) δ (ppm): 154.4, 154.1, 151.5, 150.6, 138.9, 133.8, 129.1, 127.3, 127.0, 121.7, 116.2, 114.2, 71.7, 70.4, 70.4, 70.3, 70.3, 70.3, 70.2, 69.7, 58.8, 40.9; ESI-MS (ion trap): *m*/*z* 1193 [M + H]^+^. HRMS (ESI+): *m/z* 1192.6231 [M + H]^+^, calcd for C_56_H_94_N_3_O_24_: 1192.6227.

## 4. Conclusions 

It was previously shown [47] that the *N,N*-disubstituited carbamate protecting group is too stable to regenerate resveratrol at convenient rates under physiological conditions. This work also marks progress in comparison with ref. [60]. The ether and ester bonds used in [60] were too stable or too unstable for use in prodrugs, respectively. The only carboxylic ester derivative hydrolyzing relatively slowly in plasma was the PEG–PLA copolymer conjugate which formed supramolecular structures in aqueous solution and presumably after i.v. injection [60]. How this construct would behave after oral or intragastric administration was not tested. The *N*-monosubstituted carbamate ester derivatives synthesized herein show, instead, optimal stability for use as prodrugs: high stability under acidic conditions to withstand passage through the stomach, slow hydrolysis at intestinal pH, and faster hydrolysis in blood. The selected promoieties, short monodispersed methoxy-oligo(ethylene glycol) chains, may be a convenient tool for modulating the properties and, thus, improving the performance of the resulting prodrugs.

## References

[1] Beyerle J., Frei E., Stiborova M., Habermann N., Ulrich C.M. (2015). Biotransformation of xenobiotics in the human colon and rectum and its association with colorectal cancer. Drug Metab. Rev..

[2] Bock K.W. (2014). Homeostatic control of xeno- and endobiotics in the drug-metabolizing enzyme system. Biochem. Pharmacol..

[3] Gundert-Remy U., Bernauer U., Blomeke B., Doring B., Fabian E., Goebel C., Hessel S., Jackh C., Lampen A., Oesch F. (2014). Extrahepatic metabolism at the body’s internal-external interfaces. Drug Metab. Rev..

[4] Hrycay E.G., Bandiera S.M. (2012). The monooxygenase, peroxidase, and peroxygenase properties of cytochrome P450. Arch. Biochem. Biophys..

[5] Hrycay E.G., Bandiera S.M. (2015). Monooxygenase, peroxidase and peroxygenase properties and reaction mechanisms of cytochrome p450 enzymes. Adv. Exp. Med. Biol..

[6] Dong D., Ako R., Wu B. (2012). Crystal structures of human sulfotransferases: Insights into the mechanisms of action and substrate selectivity. Exp. Opin. Drug Metab. Toxicol..

[7] Runge-Morris M., Kocarek T.A., Falany C.N. (2013). Regulation of the cytosolic sulfotransferases by nuclear receptors. Drug Metab. Rev..

[8] Oda S., Fukami T., Yokoi T., Nakajima M. (2015). A comprehensive review of UDP-glucuronosyltransferase and esterases for drug development. Drug Metab. Pharmacokinet..

[9] Ouzzine M., Gulberti S., Ramalanjaona N., Magdalou J., Fournel-Gigleux S. (2014). The UDP-glucuronosyltransferases of the bloodbrain barrier: Their role in drug metabolism and detoxication. Front. Cell Neurosci..

[10] Roth M., Obaidat A., Hagenbuch B. (2012). OATPs, OATs and OCTs: The organic anion and cation transporters of the SLCO and SLC22A gene superfamilies. Br. J. Pharmacol..

[11] Homolya L., Varadi A., Sarkadi B. (2003). Multidrug resistance-associated proteins: Export pumps for conjugates with glutathione, glucuronate or sulfate. Biofactors.

[12] Wilkens S. (2015). Structure and mechanism of ABC transporters. F1000Prime Rep..

[13] Zhou S.F., Wang L.L., Di Y.M., Xue C.C., Duan W., Li C.G., Li Y. (2008). Substrates and inhibitors of human multidrug resistance associated proteins and the implications in drug development. Curr. Med. Chem..

[14] Cottart C.H., Nivet-Antoine V., Laguillier-Morizot C., Beaudeux J.L. (2010). Resveratrol bioavailability and toxicity in humans. Mol. Nutr. Food Res..

[15] Lancon A., Hanet N., Jannin B., Delmas D., Heydel J.M., Lizard G., Chagnon M.C., Artur Y., Latruffe N. (2007). Resveratrol in human hepatoma HepG2 cells: Metabolism and inducibility of detoxifying enzymes. Drug Metab. Dispos..

[16] Lou B.S., Wu P.S., Hou C.W., Cheng F.Y., Chen J.K. (2014). Simultaneous quantification of *trans*-resveratrol and its sulfate and glucuronide metabolites in rat tissues by stable isotope-dilution UPLC-MS/MS analysis. J. Pharm. Biomed. Anal..

[17] Maier-Salamon A., Bohmdorfer M., Riha J., Thalhammer T., Szekeres T., Jaeger W. (2013). Interplay between metabolism and transport of resveratrol. Ann. N.Y. Acad. Sci..

[18] Murakami I., Chaleckis R., Pluskal T., Ito K., Hori K., Ebe M., Yanagida M., Kondoh H. (2014). Metabolism of skin-absorbed resveratrol into its glucuronized form in mouse skin. PLoS ONE.

[19] Walle T. (2011). Bioavailability of resveratrol. Ann. N.Y. Acad. Sci..

[20] Wenzel E., Soldo T., Erbersdobler H., Somoza V. (2005). Bioactivity and metabolism of *trans*-resveratrol orally administered to Wistar rats. Mol. Nutr. Food Res..

[21] Wenzel E., Somoza V. (2005). Metabolism and bioavailability of trans-resveratrol. Mol. Nutr. Food Res..

[22] Juan M.E., Gonzalez-Pons E., Planas J.M. (2010). Multidrug resistance proteins restrain the intestinal absorption of *trans*-resveratrol in rats. J. Nutr..

[23] Planas J.M., Alfaras I., Colom H., Juan M.E. (2012). The bioavailability and distribution of *trans*-resveratrol are constrained by ABC transporters. Arch. Biochem. Biophys..

[24] Riha J., Brenner S., Bohmdorfer M., Giessrigl B., Pignitter M., Schueller K., Thalhammer T., Stieger B., Somoza V., Szekeres T. (2014). Resveratrol and its major sulfated conjugates are substrates of organic anion transporting polypeptides (OATPs): Impact on growth of ZR-75-1 breast cancer cells. Mol. Nutr. Food Res..

[25] Novelle M.G., Wahl D., Dieguez C., Bernier M., de Cabo R. (2015). Resveratrol supplementation: Where are we now and where should we go?. Ageing Res. Rev..

[26] Park E.J., Pezzuto J.M. (2015). The pharmacology of resveratrol in animals and humans. Biochim. Biophys. Acta.

[27] Han G., Xia J., Gao J., Inagaki Y., Tang W., Kokudo N. (2015). Anti-tumor effects and cellular mechanisms of resveratrol. Drug Discov. Ther..

[28] Kulkarni S.S., Canto C. (2015). The molecular targets of resveratrol. Biochim. Biophys. Acta.

[29] Latruffe N., Lancon A., Frazzi R., Aires V., Delmas D., Michaille J.J., Djouadi F., Bastin J., Cherkaoui-Malki M. (2015). Exploring new ways of regulation by resveratrol involving miRNAs, with emphasis on inflammation. Ann. N.Y. Acad. Sci..

[30] Britton R.G., Kovoor C., Brown K. (2015). Direct molecular targets of resveratrol: Identifying key interactions to unlock complex mechanisms. Ann. N.Y. Acad. Sci..

[31] Bowers J.L., Tyulmenkov V.V., Jernigan S.C., Klinge C.M. (2000). Resveratrol acts as a mixed agonist/antagonist for estrogen receptors alpha and beta. Endocrinology.

[32] Chakraborty S., Levenson A.S., Biswas P.K. (2013). Structural insights into Resveratrol’s antagonist and partial agonist actions on estrogen receptor alpha. BMC Struct. Biol..

[33] Gehm B.D., McAndrews J.M., Chien P.Y., Jameson J.L. (1997). Resveratrol, a polyphenolic compound found in grapes and wine, is an agonist for the estrogen receptor. Proc. Natl. Acad. Sci. USA.

[34] Lopes Costa A., Le Bachelier C., Mathieu L., Rotig A., Boneh A., de Lonlay P., Tarnopolsky M.A., Thorburn D.R., Bastin J., Djouadi F. (2014). Beneficial effects of resveratrol on respiratory chain defects in patients’ fibroblasts involve estrogen receptor and estrogen-related receptor alpha signaling. Hum. Mol. Genet..

[35] Nwachukwu J.C., Srinivasan S., Bruno N.E., Parent A.A., Hughes T.S., Pollock J.A., Gjyshi O., Cavett V., Nowak J., Garcia-Ordonez R.D. (2014). Resveratrol modulates the inflammatory response via an estrogen receptor-signal integration network. Elife.

[36] Robb E.L., Stuart J.A. (2014). The stilbenes resveratrol, pterostilbene and piceid affect growth and stress resistance in mammalian cells via a mechanism requiring estrogen receptor beta and the induction of Mn-superoxide dismutase. Phytochemistry.

[37] Ruotolo R., Calani L., Fietta E., Brighenti F., Crozier A., Meda C., Maggi A., Ottonello S., Del Rio D. (2013). Anti-estrogenic activity of a human resveratrol metabolite. Nutr. Metab. Cardiovasc. Dis..

[38] Saleh M.C., Connell B.J., Saleh T.M. (2013). Resveratrol induced neuroprotection is mediated via both estrogen receptor subtypes, ER_α_ and ER_β_. Neurosci. Lett..

[39] Cottart C.H., Nivet-Antoine V., Beaudeux J.L. (2014). Review of recent data on the metabolism, biological effects, and toxicity of resveratrol in humans. Mol. Nutr. Food Res..

[40] Cottart C.H., Nivet-Antoine V., Beaudeux J.L. (2015). Is resveratrol an imposter?. Mol. Nutr. Food Res..

[41] Hausenblas H.A., Schoulda J.A., Smoliga J.M. (2015). Resveratrol treatment as an adjunct to pharmacological management in type 2 diabetes mellitus—Systematic review and meta-analysis. Mol. Nutr. Food Res..

[42] Nunez-Sanchez M.A., Gonzalez-Sarrias A., Romo-Vaquero M., Garcia-Villalba R., Selma M.V., Tomas-Barberan F.A., Garcia-Conesa M.T., Espin J.C. (2015). Dietary phenolics against colorectal cancer—From promising preclinical results to poor translation into clinical trials: Pitfalls and future needs. Mol. Nutr. Food Res..

[43] Smoliga J.M., Baur J.A., Hausenblas H.A. (2011). Resveratrol and health—A comprehensive review of human clinical trials. Mol. Nutr. Food Res..

[44] Visioli F. (2014). The resveratrol fiasco. Pharmacol. Res..

[45] Biasutto L., Mattarei A., Sassi N., Azzolini M., Romio M., Paradisi C., Zoratti M. (2014). Improving the efficacy of plant polyphenols. Anticancer Agents Med. Chem..

[46] Biasutto L., Zoratti M. (2014). Prodrugs of quercetin and resveratrol: A strategy under development. Curr. Drug Metab..

[47] Mattarei A., Carraro M., Azzolini M., Paradisi C., Zoratti M., Biasutto L. (2014). New Water-Soluble Carbamate Ester Derivatives of Resveratrol. Molecules.

[48] Banerjee S.S., Aher N., Patil R., Khandare J. (2012). Poly(ethylene glycol)-Prodrug Conjugates: Concept, Design, and Applications. J. Drug Deliv..

[49] Greenwald R.B., Choe Y.H., McGuire J., Conover C.D. (2003). Effective drug delivery by PEGylated drug conjugates. Adv. Drug Deliv. Rev..

[50] Kolate A., Baradia D., Patil S., Vhora I., Kore G., Misra A. (2014). PEG—A versatile conjugating ligand for drugs and drug delivery systems. J. Control. Release.

[51] Pasut G., Veronese F.M. (2009). PEGylation for improving the effectiveness of therapeutic biomolecules. Drugs Today (Barc).

[52] Pasut G., Veronese F.M. (2012). State of the art in PEGylation: The great versatility achieved after forty years of research. J. Control. Release.

[53] Veronese F.M., Pasut G. (2005). PEGylation, successful approach to drug delivery. Drug Discov. Today.

[54] Ginn C., Khalili H., Lever R., Brocchini S. (2014). PEGylation and its impact on the design of new protein-based medicines. Future Med. Chem..

[55] Mero A., Clementi C., Veronese F.M., Pasut G. (2011). Covalent conjugation of poly(ethylene glycol) to proteins and peptides: Strategies and methods. Methods Mol. Biol..

[56] Milla P., Dosio F., Cattel L. (2012). PEGylation of proteins and liposomes: A powerful and flexible strategy to improve the drug delivery. Curr. Drug Metab..

[57] Pasut G. (2014). Pegylation of biological molecules and potential benefits: Pharmacological properties of certolizumab pegol. BioDrugs.

[58] Vllasaliu D., Fowler R., Stolnik S. (2014). PEGylated nanomedicines: Recent progress and remaining concerns. Exp. Opin. Drug Deliv..

[59] Zundorf I., Dingermann T. (2014). PEGylation—A well-proven strategy for the improvement of recombinant drugs. Pharmazie.

[60] Siddalingappa B., Benson H.A.E., Brown D.H., Batty K.T., Chen Y. (2015). Stabilization of Resveratrol in Blood Circulation by Conjugation to mPEG and mPEG-PLA Polymers: Investigation of Conjugate Linker and Polymer Composition on Stability, Metabolism, Antioxidant Activity and Pharmacokinetic Profile. PLoS ONE.

[61] Mattarei A., Azzolini M., Carraro M., Sassi N., Zoratti M., Paradisi C., Biasutto L. (2013). Acetal derivatives as prodrugs of resveratrol. Mol. Pharm..

[62] Kozerski G.E., Gallavan R.H., Ziemelis M.J. (2003). Investigation of trialkoxysilane hydrolysis kinetics using liquid chromatography with inductively coupled plasma atomic emission spectrometric detection and non-linear regression modeling. Anal. Chim. Acta.

[63] Adams P., Baron F.A. (1965). Esters of Carbamic Acid. Chem. Rev..

[64] Dittert L.W., Higuchi T. (1963). Rates of hydrolysis of carbamate and carbonate esters in alkaline solution. J. Pharm. Sci..

[65] Biasutto L., Marotta E., Garbisa S., Zoratti M., Paradisi C. (2010). Determination of quercetin and resveratrol in whole blood—Implications for bioavailability studies. Molecules.

[66] Biasutto L., Marotta E., Bradaschia A., Fallica M., Mattarei A., Garbisa S., Zoratti M., Paradisi C. (2009). Soluble polyphenols: Synthesis and bioavailability of 3,4′,5-tri(α-d-glucose-3-*O*-succinyl) resveratrol. Bioorg. Med. Chem. Lett..

